# Spondin 2 promotes the proliferation, migration and invasion of gastric cancer cells

**DOI:** 10.1111/jcmm.14618

**Published:** 2019-11-05

**Authors:** Haoming Lu, Ying Feng, Yilin Hu, Yibing Guo, Yifei Liu, Qinsheng Mao, Wanjiang Xue

**Affiliations:** ^1^ Department of Gastrointestinal Surgery Nantong University Affiliated Hospital Nantong China; ^2^ Research Center of Clinical Medicine Nantong University Affiliated Hospital Nantong China; ^3^ Department of Pathology Nantong University Affiliated Hospital Nantong China

**Keywords:** ERK1/2, gastric cancer, metastasis, proliferation, *SPON2*

## Abstract

Spondin 2 (SPON2), a member of the Mindin F‐Spondin family, identifies pathogens, activates congenital immunity and promotes the growth and adhesion of neurons as well as binding to their receptors, but its role in promoting or inhibiting tumour metastasis is controversial. Here, we investigated its expression levels and mechanism of action in gastric cancer (GC). Western blotting and GC tissue arrays were used to determine the expression levels of SPON2. ELISAs were performed to measure the serum levels of SPON2 in patients with GC. Two GC cell lines expressing low levels of SPON2 were used to analyse the effects of regulating SPON2 expression on proliferation, migration, invasion, the cell cycle and apoptosis. The results revealed that SPON2 was highly expressed in GC tissues from patients with relapse or metastasis. The levels of SPON2 in sera of patients with GC were significantly higher compared with those of healthy individuals and patients with atrophic gastritis. Knockdown of *SPON2* expression significantly inhibited the proliferation, migration and invasion of GC cells in vitro and in vivo. Down‐regulation of *SPON2* arrested the cell cycle in G1/S, accelerated apoptosis through the mitochondrial pathway and inhibited the epithelial‐mesenchymal transition by blocking activation of the ERK1/2 pathway. In summary, this study suggests that SPON2 acts as an oncogene in the development of GC and may serve as a marker for the diagnosing GC as well as a new therapeutic target for GC.

## 1 INTRODUCTION

Gastric cancer (GC) is the fifth most common malignant tumour worldwide, with approximately 1 033 701 new cases diagnosed in 2018.^1^ Most patients with GC present with progressive disease that may rapidly generate peritoneal and haematogenous metastases. The prognosis of patients with GC is poor,^2^, ^3^, ^4^, ^5^ and it is therefore important to determine the mechanisms of pathogenesis and metastasis to identify new molecular markers for early diagnosis as well as new therapeutic targets.

Secreting‐type ECM spondin 2 (*SPON2*, formerly Mindin, DIL‐1) is a member of the Mindin F‐Spondin family. Human *SPON2* comprises 331 amino acid residues (36 kD).^6^, ^7^ *SPON2* expression identifies pathogens, activates congenital immunity and promotes the growth and adhesion of neurons as well as binding to their receptors.^8^ *SPON2* inhibits myocardial hypertrophy through the AKT‐GSK3β and TGF‐β1–SMAD signal transduction pathways.^9^, ^10^

*SPON2* is highly expressed in numerous tumours, and elevated serum levels of *SPON2* serve as a marker for prostate and ovarian cancers.^11^, ^12^ However, the physiological function of *SPON2* in tumours and its associated molecular mechanisms are controversial. For example, *SPON2* promotes the infiltration of M1‐like macrophages by affecting the activity of the RhoA‐Rho kinase signalling pathway, further inhibiting hepatocellular carcinoma (HCC) cells from invading adjacent tissues and migrating to distant sites. Further, this process is positively regulated by thyroid hormones, which is exploited to improve the prognosis of patients with HCC.^13^ However, other studies show that high levels of *SPON2* are associated with poor prognosis of patients with prostate, hepatocellular and lung cancers.^14^, ^15^, ^16^ In colon cancer, *SPON2* acts as a downstream effector of the product of the metastasis‐associated gene 1. Moreover, overexpression of *SPON2* enhances the proliferation, migration, invasion and colony formation by colorectal cancer cells and induces metastasis to the liver.^17^

Studies of small numbers of patients with GC show that overexpression of *SPON2* is associated with poor prognosis, although the underlying mechanisms and their effects on GC cells are unknown.^18^ Therefore, the current study employed a larger number of patients to answer these questions. Here we show that high expression of *SPON2* was associated with poor prognosis of GC and may therefore serve as an auxiliary serological marker for early diagnosis and to evaluate the efficacy of treatments for GC. Moreover, down‐regulation of *SPON2* expression promoted apoptosis of GC cells and inhibited their abilities to invade and migrate by blocking activation of the ERK1/2 pathway.

## 2 MATERIALS AND METHODS

### 2.1 Patients, tissue samples and blood samples

Patients with GC, gastric stromal tumours and atrophic gastritis were definitively diagnosed according to pathological data. Tissue samples and blood samples were provided by the Affiliated Hospital of Nantong University. Patients granted their informed consent before the study commenced. Matched pairs of GC tissues and para‐tumorous normal mucosal tissue samples (n = 207) used to construct tissue chips were acquired from patients with GC who underwent radical surgery from January through December of 2010. Complete medical and follow‐up data were available for all patients.

Overall survival (OS) and disease‐free survival (DFS) refer to the interval from the time of surgery to death or recurrence, respectively. The cut‐off for postoperative follow‐up visits was June 2015 (median follow‐up 60 months; range, 2‐60 months). Patients did not harbour detectable distant metastasis or malignant tumours in other sites upon preoperative examination, and they were not administered neoadjuvant chemotherapy, radiotherapy, immunotherapy or other specialized treatment.

We analysed tissues from patients with T3N0M0 GC (n = 20) (patients who did not experience recurrence or metastasis 3 years after surgery [n = 12] and patients who relapsed and experienced metastasis within 3 years after surgery [n = 8]). We collected serum samples (n = 43) from patients with GC before and after radical surgery (without preoperative neoadjuvant chemotherapy and conversion therapy), serum samples (n = 20) before and after three cycles of neoadjuvant chemotherapy with SOX regimen and serum samples (n = 20) before and after three cycles of conversion therapy with SOX regimen.

We performed enhanced computed tomography (CT) of the abdomen to determine the efficacy of three complete cycles of chemotherapy. The objective response rate of patients who underwent neoadjuvant chemotherapy and conversion therapy was evaluated according to the Response Evaluation Criteria in Solid Tumors (RECIST1.1) as follows: complete response (CR), partial response (PR), stable disease (SD) or progressive disease (PD).^19^ Serum samples from 25 healthy volunteers, 20 patients with atrophic gastritis and 20 patients with gastric stromal tumours were provided during the course of the study. The Human Research Ethics Committee of Affiliated Hospital of Nantong University approved these procedures.

### 2.2 Cell culture, viral transfection and plasmid transfection

A human cell line derived from normal gastric mucosa (GES‐1) and the GC cell lines SGC‐7901, BGC‐823, AGS, MKN‐27, MKN‐28 and MGC‐803 were purchased from GeneChem. Cell lines were cultured in RPMI‐1640 medium supplemented with 10% foetal bovine serum (FBS) and 100 µg/mL penicillin‐streptomycin in an atmosphere containing 5% CO_2_ at 37°C. To generate GC cell lines stably expressing low levels of *SPON2*, we transfected GC cells with a lentivirus vector encoding a *SPON2*‐specific short hairpin RNA‐expressing (GeneChem) and a lentivirus encoding a random sequence of shRNA (negative control). The activities of the constructs were verified using Western blotting. The sh*SPON2* sequence was 5′‐CCGGCAGGGACAATGAGATTGTAGACTCGAGTCTACAATCTCATTGTCCCTGTTTTTG‐3′. *SPON2* ectopic expression and negative control lentiviruses were also purchased from GeneChem, and the overexpressing cells were selected and used for next experiments. We transfected GC cell lines with pcDNA3.1A (−) CCND1 from Dr Xiong Yu (University of North Carolina Lineberger Comprehensive Cancer Center) and pcDNA‐ERK1/2 (GeneChem) in the presence of Lipofectamine 2000 (Invitrogen), according to the manufacturer's instructions. We verified the expression of each insert using Western blotting 48 hours before performing subsequent experiments.

### 2.3 RNA extraction and real‐time quantitative PCR (qRT‐PCR)

We extracted total RNA from tissues and cells using TRIzol (Invitrogen) and performed qRT‐PCR as previously described.^20^, ^21^ The primer sequences used in this study were as follows:

*SPON2* forward: 5′‐AAGAACCAGTACGTCAGTATCGG‐3′;
*SPON2* reverse: 5′‐CACAAACGAGACCAGCGAGT‐3′;
*CCND1* forward: 5′‐CGAGGAACAGAAGTGCG‐3′;
*CCND1* reverse: 5′‐GGCGGTAGTAGGACAGGA‐3′;
*GAPDH* forward: 5′‐TGCACCACAACTGCTTAGC‐3′;
*GAPDH* reverse: 5′‐GGCATGGACTGTGGTCATGAG‐3′.

### 2.4 Western blotting

Western blotting was performed as previously described.^22^ We used the antibodies according to the manufacturers' instructions. The primary antibodies were as follows: β‐actin mouse monoclonal (60008‐1‐Ig), *SPON2* rabbit polyclonal (20513‐1‐AP), ERK1/2 rabbit polyclonal (16443‐1‐AP), JNK rabbit polyclonal antibody (51151‐1‐AP), p38 rabbit polyclonal (14064‐1‐AP), vimentin rabbit polyclonal (10366‐1‐AP), N‐cadherin rabbit polyclonal (22018‐1‐AP), E‐cadherin rabbit polyclonal (20874‐1‐AP; all from Proteintech Group, Inc), BAX rabbit polyclonal (#5023), BCL‐2 rabbit polyclonal (#9942), cleaved caspase‐3 rabbit polyclonal (#9664), cleaved PARP mouse polyclonal (#5625), p‐ERK1/2 rabbit polyclonal (#4370; all from Cell Signaling Technology), p‐p38 (D‐8; sc‐7973) and p‐JNK antibody (G‐7; sc‐6254; both from Santa Cruz Biotechnology, Inc).

### 2.5 Tissue microarray (TMA) construction and immunohistochemical analysis

All tested tissues were fixed with 4% polyoxymethylene for 1 hour, dehydrated through an ethanol gradient and embedded in paraffin. TMA and xenograft tissue samples were incubated with antibodies after deparaffinization, rehydration, antigen retrieval and quenching of endogenous peroxidase. The slides were incubated overnight with primary antibodies against *SPON2* (1:500, Proteintech, 20513‐1‐AP), E‐cadherin (1:100, Proteintech, 20874‐1‐AP), N‐cadherin (1:100, Proteintech, 22018‐1‐AP), Vimentin (1:100, Proteintech, 10366‐1‐AP), p‐ERK1/2 (1:100, Cell Signaling Technology, 4370) and ERK1/2 (1:100, Proteintech, 16443‐1‐AP) at 4°C. Two pathologists evaluated the IHC results using the criteria as follows: 0 (negative), 1 (weak positive), 2 (moderate positive) and 3 (strong positive). The positive rate was evaluated by assigning the grades as follows: 0 (<5%), 1 (5%‐25%), 2 (>25%‐50%), 3 (>50%‐75%) and 4 (>75). The final expression score of a target protein was calculated by multiplying the positive score (percentage) by the staining intensity score. The staining scores were defined as follows: high expression (score ≥ 3) and low expression (score ≤ 2).

### 2.6 ELISA

Serum samples were stored at −80°C. A Human *SPON2* ELISA kit was purchased from Jijin Chemistry Technology Co. The concentration of *SPON2* in serum was determined using the standard protein sample provided with the kit. A receiver operator characteristic (ROC) curve was plotted to calculate the ROC area under the curve (AUC) and to compare the sensitivity and specificity of serum levels of *SPON2* in early‐stage GC.

### 2.7 Cell proliferation and colony‐forming assays

To measure proliferation, cells (5 × 10^3^ GC cells per well) were added to a 96‐well plate and cells were cultured for 0, 1, 2, 3 and 4 days. A Cell Counting Kit‐8 kit (CCK‐8; Beyotime Institute of Biotechnology) was used to measure cell proliferation. To measure colony formation, we added 200 GC cells per well to a 6‐well plate, followed by culture for 14 days. Cells were fixed with 4% paraformaldehyde, stained with 1% crystal violet, and colonies comprising >50 cells were counted.

### 2.8 Flow cytometry

Gastric cancer cells (5 × 10^5^ GC cells per well) were added to a 6‐well plate. Cells were harvested after 24 hours and fixed overnight with 70% ethyl alcohol. The cells were then incubated in a solution containing propidium iodide (0.5 mg/mL) and RNase A (10 mg/mL; BD Biosciences Pharmingen) for 20 minutes after washing. A flow cytometer (BD Biosciences Pharmingen) was used to determine DNA content. The rate of apoptosis was measured as previously described.^23^ Cells (1 × 10^6^) were stained with annexin V‐PE and 7‐AAD (BD Biosciences).

### 2.9 Mitochondrial membrane potential

Gastric cancer cells were treated with JC‐1 (5 µg/mL) to measure mitochondrial membrane potential (JC‐1; YEASEN) according to the manufacturer's instructions.^24^ The red and green fluorescence intensities of GC cells were observed using an inverted fluorescence microscope (TE‐2000S, Nikon).

### 2.10 Wound healing, Transwell and cell adhesion assays

For wound healing assays, cells (5 × 10^5^ per well) were added to a 6‐well plate, and the monolayers were scratched with a 200‐µL pipette tip. Wound healing was observed at 0 hour and 48 hours, and images were captured using an inverted microscope (TE‐2000S, Nikon). In the invasion analysis, we added diluted Matrigel to the wells of a Transwell plate, followed by the addition of 1 × 10^5^ cells in 200‐µL serum‐free medium to the upper chamber and 500‐µL culture medium containing 20% FBS to the lower chamber. After 24 hours, cells were fixed with 4% paraformaldehyde and stained with 1% crystal violet. The cells in five randomly selected visual fields were counted. In cell adhesion assays, 10 μg/mL fibronectin (Solarbio) was used to coat a 96‐well plate, which was sealed with 1% bovine serum albumin and then seeded with 3 × 10^4^ cells per well. Cells were stained with 10% crystal violet, and an inverted fluorescence microscope was used to count the adherent cells in five randomly selected visual fields.

### 2.11 Luciferase assays

To detect cyclin D1 (*CCND1*) promoter activity, we co‐transfected human embryonic kidney cells HEK293T cells (in the presence of Lipofectamine 2000) with pRL‐TK (Promega), PD1Luc from Myriam Gorospe and Heiko Muller, respectively) and the *SPON2* eukaryotic expression vector pcDNA3.1(+) *SPON2* (GeneChem). After 24 hours, cells were analysed using a Dual‐luciferase Reporter Assay Kit (Beyotime Institute of Biotechnology) according to the manufacturer's instructions. Luciferase activity was expressed as the ratio of the fluorescence intensities of the test to control cells.^25^

### 2.12 Mouse experiments

We developed a nude mouse model of subcutaneous tumorigenicity as follows: male 1‐month‐old nude BALB/c mice (n = 6 per group), purchased from the Animal Center of Medical College of Nantong University, were subcutaneously injected with 5 × 10^6^ GC cells into the right side of the back. Tumours were measured every 5 days, and gross tumour volume was calculated as follows: V (volume, mm^3^) = 0.5 × L (length, mm) × W^2^ (width, mm^2^). Mice were killed after 5 weeks, and subcutaneous tumours were excised and weighed. To develop a nude mouse model of peritoneal metastasis, 6 × 10^6^ GC cells were injected into the abdominal cavity of 1‐month‐old male BALB/c nude mice (n = 4/group). Mice were killed after 4 weeks, and then their abdominal cavities were inspected to calculate the quantity of transplanted tumours. The Animal Care Committee of Nantong University reviewed and approved these experiments.

### 2.13 Statistical analysis

Data are expressed as the mean ± standard deviation (SD), and statistical analysis was performed with SPSS 22.0. *P* < .05 indicates a significant difference. For continuous variables, differences between two groups were evaluated using the chi‐square test, *t* test or one‐way analysis of variance. Spearman's correlation rank analysis was used to analyse categorical variables. Logistic regression analysis was performed to identify risk factors affecting *SPON2* levels in GC tissues. The Kaplan‐Meier method was applied to calculate the survival rates of patients with GC. OS and DFS were evaluated using the log‐rank test, and prognostic factors were evaluated using the Cox regression model.

## 3 RESULTS

### 3.1 *SPON2* overexpression is associated with poor prognosis of patients with GC

**Figure 1:**
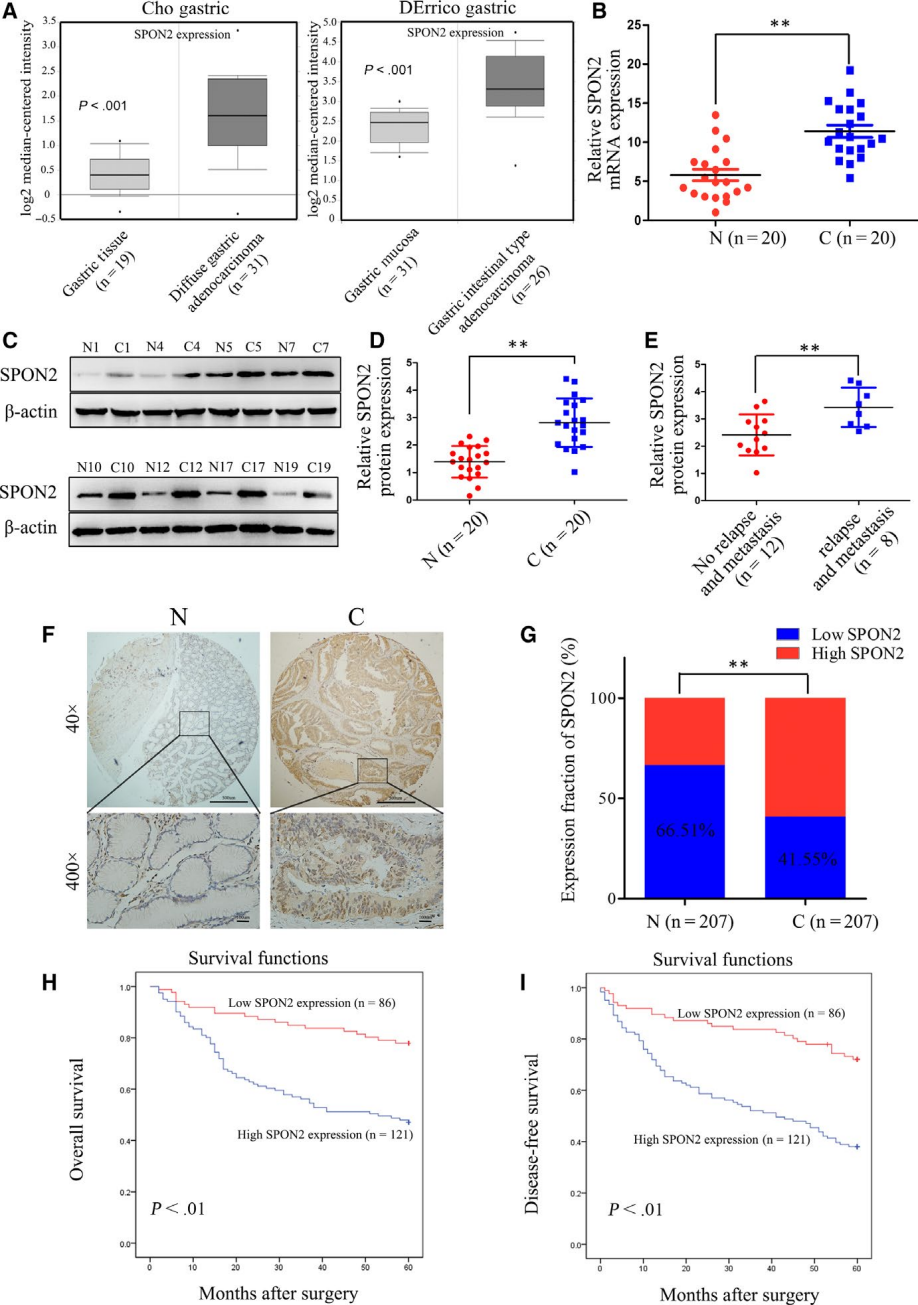
SPON2 is overexpressed in GC tissues. A, *SPON2* expression is higher in GC tissues than para‐tumorous tissues according to ONCOMINE analysis (Cho Gastric and DErrico Gastric, all *P* < .001). B‐D, qRT‐PCR and Western blotting analyses of *SPON2* mRNA and protein expression in 20 matched pairs of T3N0M0 GC tissues (C) and para‐tumorous tissues (N). Data represent the mean ± SD of three independent experiments, **P < .01. E, Western blot analysis of SPON2 expression in eight GC tissue samples acquired from patients with relapse or metastasis 3 y after surgery for GC and in 12 samples acquired from patients without relapse or metastasis, *P < .05. F, Representative images of IHC analysis of SPON2 expression in 207 matched pairs of GC tissues and para‐tumorous tissues (upper: magnification ×40; lower: magnification ×400). G, IHC analysis of SPON2 expression in 207 matched pairs of GC tissue samples and para‐tumorous tissues, **P < .01. H, I, Kaplan‐Meier survival analysis of 207 GC patients with low or high *SPON2* expression. Patients with high SPON2 expression experienced longer overall survival (OS) and disease‐free survival (DFS) after surgery

We first analysed *SPON2* expression using the ONCOMINE cancer microarray database, which revealed that *SPON2* levels in GC tissues are higher compared with those in para‐tumorous tissues (Figure 1A). Western blotting and qRT‐PCR analyses revealed that the levels of *SPON2* in 20 T3N0M0 GC tissues were higher compared with those of the corresponding para‐tumorous tissues (Figure 1B,C). Further, *SPON2* levels in eight cancer tissue samples of patients with relapse or metastasis were significantly higher compared with those without relapse or metastasis (Figure 1D).

To analyse the associations between *SPON2* levels and clinicopathological features of patients with GC, we used IHC to analyse 207 pairs of GC tissue samples on a tissue chip (Figure 1E). Although we detected the expression of *SPON2* in both GC cells and stroma, we found that *SPON2* was mainly expressed in GC cells. Moreover, the purpose of this study is to observe the effect of *SPON2* expression of GC cells on their own biology. Therefore, we only observed the expression of *SPON2* in GC cells during IHC detection. The levels of *SPON2* in GC tissue were significantly higher compared with those in para‐tumorous tissue (Figure 1F). Univariate analysis revealed that high levels of *SPON2* were significantly associated with differentiation, T‐ and N‐staging but not significantly associated with age, sex, tumour site or tumour size (Table 1). Logistic regression multivariate analysis revealed that high levels of *SPON2* in GC tissues were significantly associated with N‐staging (*P* < .001, odds ratio [OR] = 4.968, 95% confidence interval [CI]: 2.544‐9.700).

**Table 1:** Correlation between SPON2 expression in GC tissues and clinicopathological features of GC patients

Clinicopathological parameter	N	SPON2		*P* value
		Low	High	
Gender				.425
Male	138	60 (43.5%)	78 (56.5%)	
Female	69	26 (37.7%)	43 (62.3%)	
Age (y)				.474
≤55	39	14 (35.9%)	25 (64.1%)	
>55	168	72 (42.9%)	96 (57.1%)	
Differentiation				.018
Well	25	16 (64.0%)	9 (36.0%)	
Moderate/poor	182	70 (38.5%)	112 (61.5%)	
Tumour diameter (cm)				.069
<4	100	48 (48.0%)	52 (52.0%)	
≥4	107	38 (35.5%)	69 (64.5%)	
Tumour localization				.388
Up	25	8 (32.0%)	17 (68.0%)	
Middle/down	182	78 (42.9%)	104 (57.1%)	
TNM stages				<.001
I/II	119	66 (55.5%)	53 (44.5%)	
III	88	20 (22.7%)	68 (77.3%)	
Depth of invasion				<.001
T1/T2	90	51 (56.7%)	39 (43.3%)	
T3/T4	117	35 (29.7%)	82 (70.3%)	
Lymph node metastasis				<.001
Negative	102	63 (61.8%)	39 (38.2%)	
Positive	105	23 (21.9%)	82 (78.1%)	

Kaplan‐Meier survival analysis revealed that postoperative OS and DFS of patients GC with high levels of *SPON2* were significantly lower compared with those with low levels (Figure 1G). Multivariate analysis revealed that a high level of *SPON2* was an independent risk factor for postoperative OS and DFS (Table 2).

**Table 2:** Univariate and multivariable analysis of OS and DFS of patients with GC

Variable	OS			DFS		
	Univariate analysis *P* > |*z*|	Multivariable analysis		Univariate analysis *P* > |*z*|	Multivariable analysis	
		*P* > |*z*|	HR (95% CI)		*P* > |*z*|	HR (95% CI)
Gender						
Male (n = 138) vs female (69)	.455			.292		
Age (y)						
≤55 (n = 39) vs >55 (n = 168)	.312			.106		
Differentiation						
Well (n = 25) vs moderate/poor (n = 182)	.096			.063		
Tumour diameter (cm)						
<4 (n = 100) vs ≥4 (107)	.314			.207		
Tumour localization						
Up (n = 25) vs middle/down (182)	.818			.984		
Depth of invasion						
T1/T2 (n = 90) vs T3/T4 (n = 117)	.002	<.001	2.843 (1.594‐5.071)	.046	.018	1.769 (1.101‐2.844)
Lymph node metastasis						
Negative (n = 102) vs positive (n = 105)	.001	.001	2.458 (1.425‐4.240)	<.001	<.001	2.779 (1.701‐4.541)
SPON2 expression						
Low (n = 86) vs high (n = 121)	.008	.013	1.952 (1.148‐3.318)	.002	.003	2.053 (1.274‐3.306)

### 3.2 Serum *SPON2* levels are increased in patients with GC

**Figure 2:**
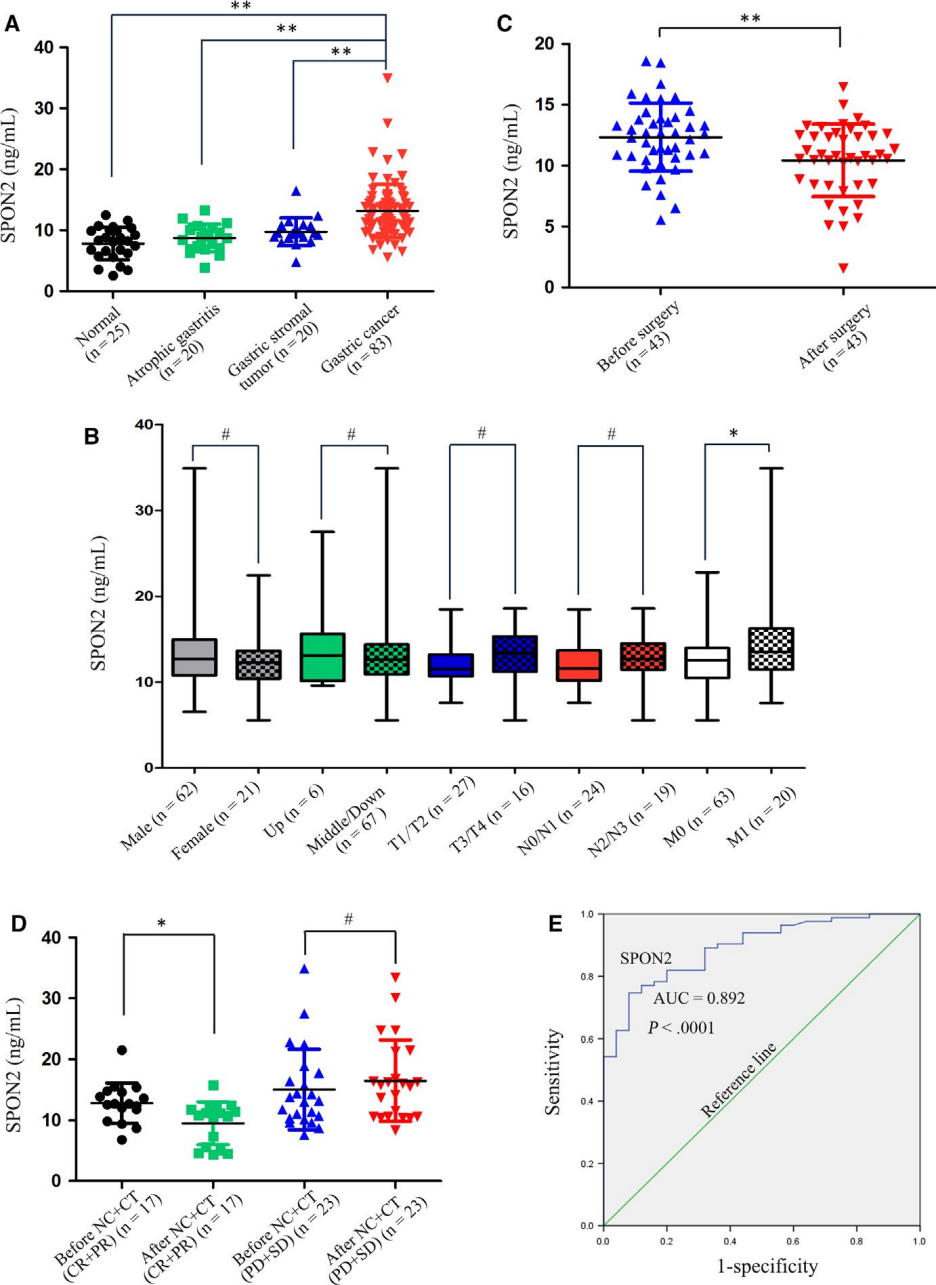
Serum SPON2 levels are up‐regulated in patients with GC. A, ELISA analysis of serum levels of SPON2 in patients with GC (n = 83), healthy controls (n = 25), patients with atrophic gastritis (n = 20) and patients with gastric stromal tumours (n = 20). Data represent the mean ± SD, **P < .01. B, Associations between serum levels of SPON2 with sex, tumour site, T stage, N stage, and M stage (M0, no liver or lung metastasis; M1, liver or lung metastasis), *P < .05, #*P* > .05. C, Changes in the serum levels of SPON2 before and after surgery in 43 patients with GC, **P < .01. D, Serum levels of SPON2 in patients with complete response and partial response (CR + PR, n = 17) and stable disease and progressive disease (SD + PD, n = 23) were evaluated using computed tomography, before and after three cycles of neoadjuvant chemotherapy or conversion therapy (NC + CT), *P < .05, #*P* > .05. E, ROC curve analysis of serum levels of SPON2 in patients with GC

To analyse whether serum *SPON2* levels can be used as a marker of GC, we analysed sera acquired from patients with different gastric lesions. Serum levels of *SPON2* in patients with GC (n = 83) were significantly higher compared with those of healthy controls (n = 25), patients with atrophic gastritis (n = 20) and patients with gastric stromal tumours (n = 20; Figure 2A), but there was no difference in serum *SPON2* levels among healthy controls, patients with atrophic gastritis and patients with gastric stromal tumours (*P* > .05). Pearson's correlation analysis indicated that there was a positive correlation between the *SPON2* expression levels of the serum and that of the GC tissue (*r* = .750, *P* < .01). Serum levels of *SPON2* were not significantly associated with sex, tumour site, and T‐ and N‐staging. However, serum levels of *SPON2* in patients with advanced GC (n = 20) with liver or lung metastasis were significantly higher compared with those without such metastases (n = 63; Figure 2B).

Spearman's rank correlation analysis revealed that serum levels of *SPON2* were significantly increased (*R* = .621, *P* < .001) in association with disease progression (healthy → non‐metastatic GC → metastatic GC). Moreover, we detected changes in serum levels of *SPON2* before and after surgery in 43 patients with GC who underwent radical surgery. Furthermore, there was a significant decrease in *SPON2* levels 1 week after surgery compared with those before surgery (Figure 2C). When we compared the serum levels of *SPON2* before and after three cycles of chemotherapy administered to patients with GC who received neoadjuvant chemotherapy (n = 20) and conversion therapy (n = 20), we found that the levels in responders (CR + PR) to neoadjuvant chemotherapy and conversion therapy were significantly lower, while those in non‐responders (SD + PD) to neoadjuvant chemotherapy and conversion therapy were not significantly increased (Figure 2D).

Receiver operator characteristic curve analysis revealed that serum levels of *SPON2* differentiated patients with GC from healthy individuals (AUC = 0.892; 95% CI, 0.829‐0.954; *P* < .001; cut‐off value for diagnosis of GC, 10.52; diagnostic sensitivity below the cut‐off, 77.1%; and specificity, 84%; Figure 2E). The corresponding positive and negative predictive values were 80.722% (95% CI, 70.29%‐88.25%) and 84% (95% CI, 63.08%‐94.74%), respectively.

### 3.3 Down‐regulation of *SPON2* expression inhibits the proliferation of GC cells

**Figure 3:**
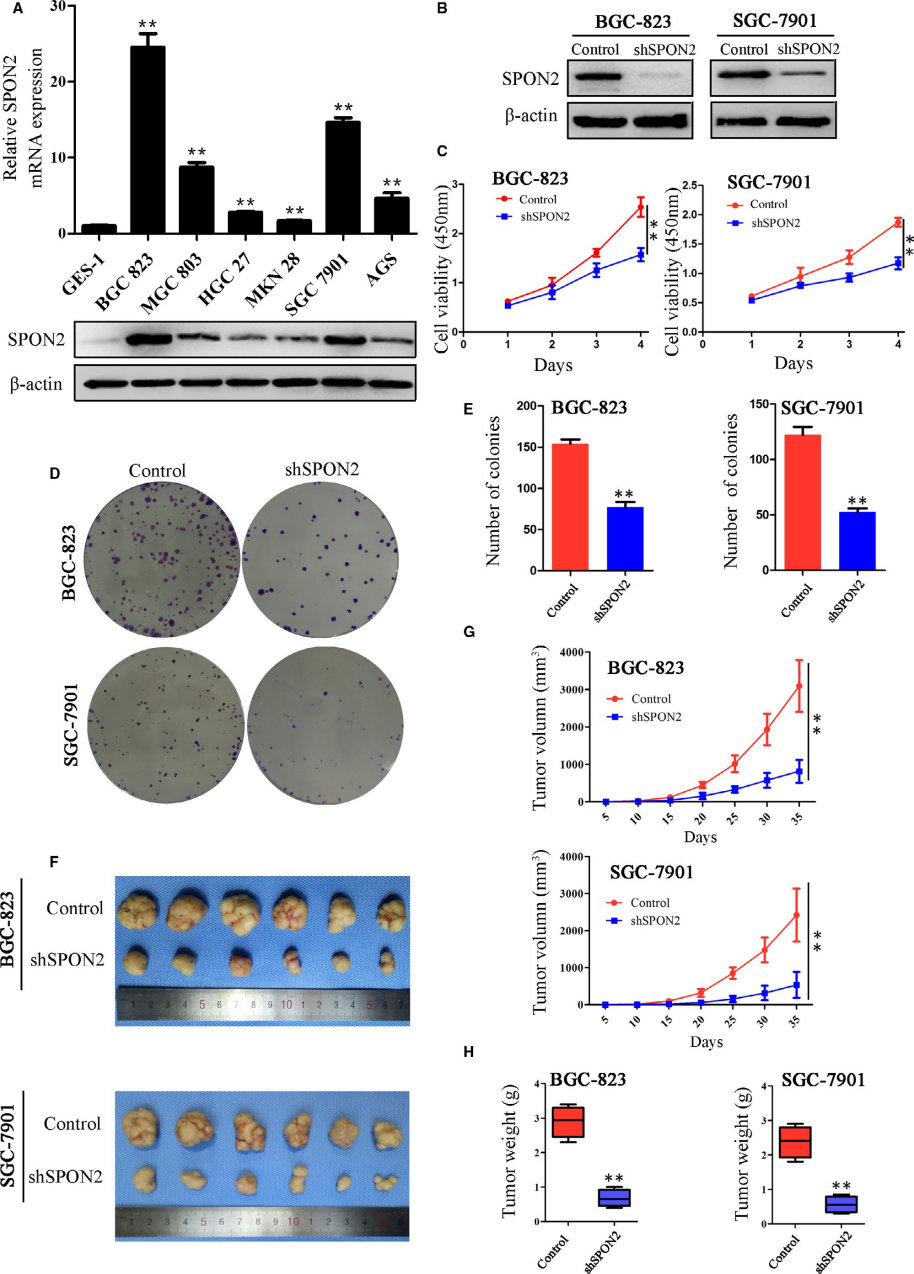
Down‐regulation of SPON2 expression inhibits the proliferation of GC cells in vitro and in vivo. A, qRT‐PCR and Western blotting analyses of SPON2 expression in GC cell lines and normal gastric cells (GES‐1). Data represent the mean ± SD of three independent experiments, *P < .05, **P < .01 compared with GES‐1 cells. B, Western blot analysis of SPON2 levels in lentivirus vector‐infected BGC‐823 and SGC‐7901 cell lines. *SPON2* expression was stably knocked down in parental lines transfected with shSPON2; control cells transfected with the ‘empty’ lentivirus (Control). C, CCK8 assay of the proliferation rates of recombinant BGC‐823 and SGC‐7901 cell lines and control cell lines, **P < .01. D, E, Colony formation assays were performed to detect the effects of SPON2 on the proliferation of recombinant BGC‐823 and SGC‐7901 cell lines and control cells. Data represent the mean ± SD of three independent experiments, **P < .01. F, G, H, Sizes, volumes and weights of tumours from mice injected with recombinant BGC‐823 and SGC‐7901 cell lines. Data represent the mean ± SD (n = 6), **P < .01

To identify the mechanism through which *SPON2* contributes to the progression of GC, we determined the levels of *SPON2* in GC cells using qRT‐PCR and Western blotting. *SPON2* mRNA and protein levels in the GC cell lines MKN‐27, MKN‐28, MGC‐803, BGC‐823, SGC‐7901 and AGS were significantly higher compared with those of GES‐1, with the levels highest in BGC‐823 and SGC‐7901 cells (Figure 3A).

We used *SPON2*‐lentivirus expression vectors to generate cell lines that stably expressed low levels of *SPON2* (BGC‐823‐sh*SPON2* and SGC‐7901‐sh*SPON2* knockdown cells; Figure 3B). Inhibition of *SPON2* expression significantly inhibited the proliferation of GC cells (Figure 3C). Colony formation assays demonstrated that knockdown of *SPON2* expression significantly decreased the number of colonies formed by each cell line (Figure 3D,E). In addition, the *SPON2*‐overexpressing lentivirus was transfected into both GES‐1 cells and AGS cells, and the transfection efficiency was monitored by Western blotting (Figure A). Overexpression of *SPON2* increases the proliferation of these two cell lines (Figure B‐D).

Consistent with these results, we found that the growth rates of subcutaneous tumours generated by the *SPON2* knockdown cell lines were lower compared with those of the controls (Figure 3F). The volumes and weights of the subcutaneous tumours after 5 weeks were significantly reduced compared with those of the control groups (Figure 3G,H).

### 3.4 Inhibition of *SPON2* expression inhibits the migration and invasiveness of GC cells in vivo and in vitro

**Figure 4:**
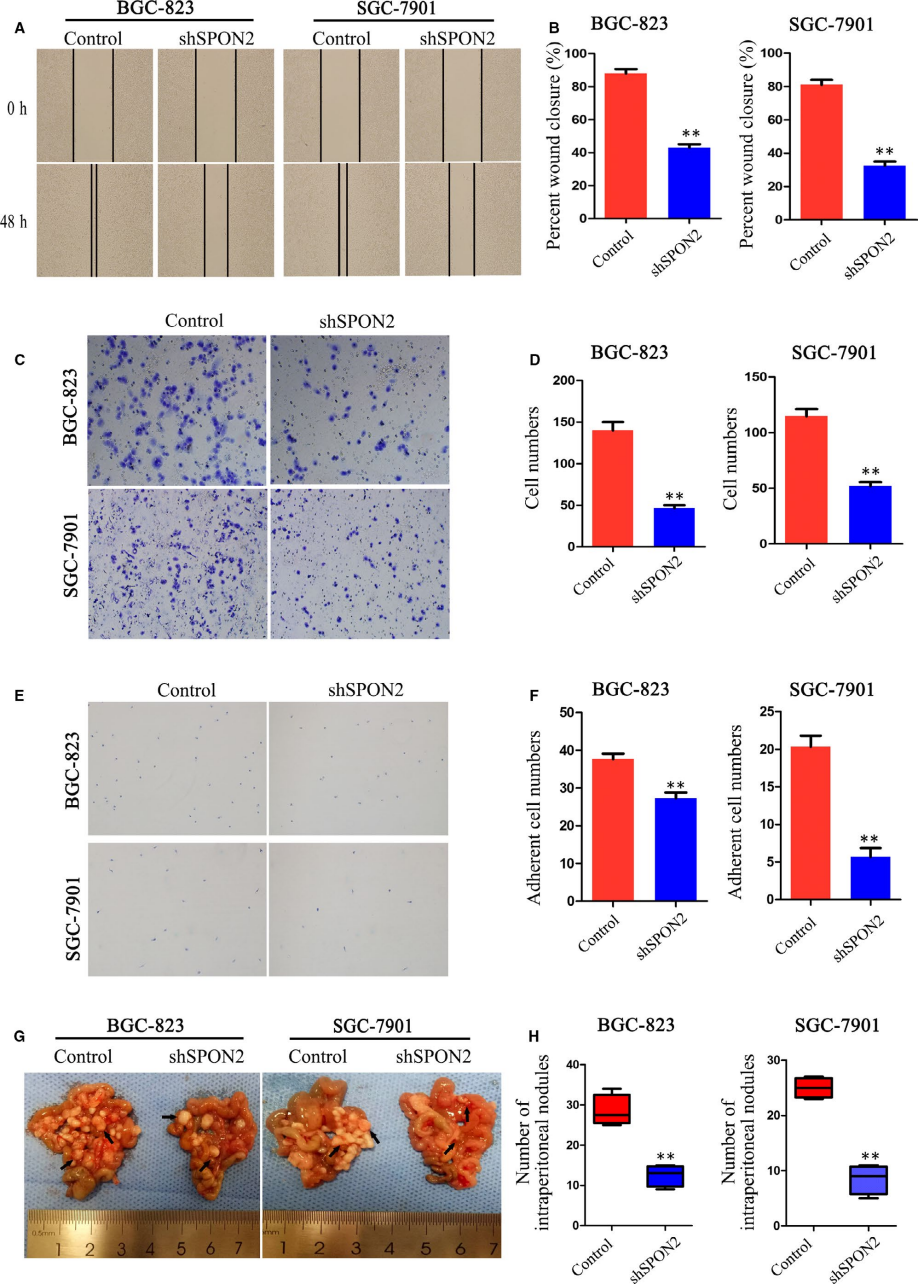
Down‐regulation of SPON2 inhibits the migration and invasion of GC cells in vitro and in vivo. A, B, Wound healing. C, D, Transwell invasion assays were performed to detect the migration and invasiveness of recombinant BGC‐823 and SGC‐7901 cell lines and control cells. Data represent the mean ± SD of three independent experiments, **P < .01. E, F, Cell adhesion assays were performed to evaluate the adhesiveness of recombinant BGC‐823 and SGC‐7901 cell lines and control cells. Data represent the mean ± SD of three independent experiments, **P < .01. G, H, Representative images and numbers of intraperitoneal metastasis in nude mice injected with recombinant BGC‐823 and SGC‐7901 cell lines and control cells, **P < .01

To analyse the mechanism through which *SPON2* contributes to metastasis and the relapse of GC, we analysed the influence of *SPON2* levels on the migration and invasiveness of GC cells. Wound healing (Figure 4A,B) and invasion assays (Figure 4C,D) showed that the ability of the *SPON2‐*knockdown cells to migrate and penetrate Matrigel was significantly reduced compared with those of the controls. Moreover, the adhesiveness of the *SPON2*‐knockdown cells was significantly lower compared with that of the controls (Figure 4E,F). Overexpression of *SPON2* in GES‐1 cells and AGS cells enhances their migration and invasion ability (Figure E‐H).

Using a nude mouse model of abdominal metastasis, we found that the number of GC cell deposits (BGC‐823 and SGC‐7901) in the abdominal cavities of nude mice engrafted with *SPON2* knockdown cells after 4 weeks was significantly lower compared with that of the controls (Figure 4G,H).

### 3.5 Down‐regulation of *SPON2* promotes G_1_ arrest

**Figure 5:**
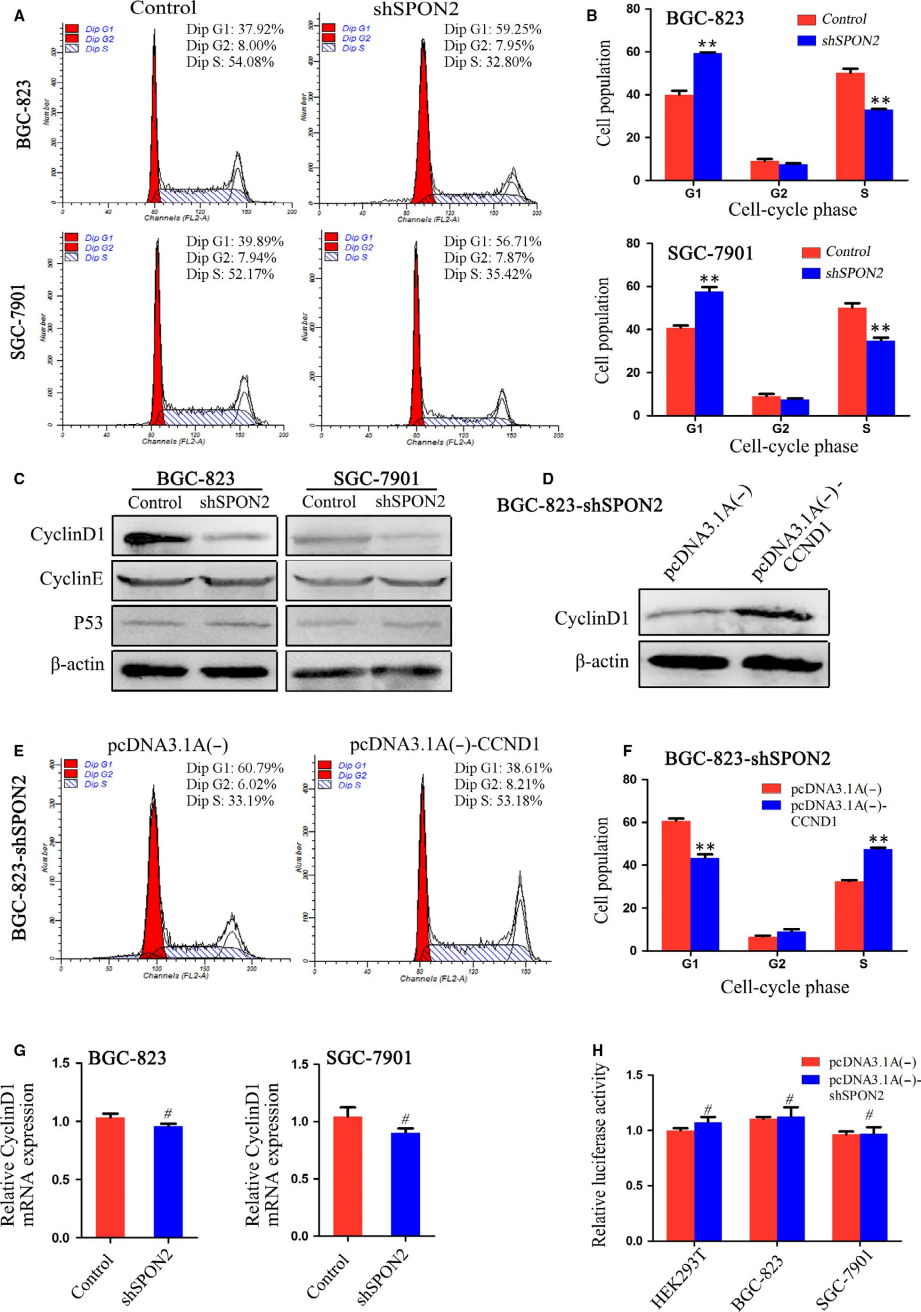
Down‐regulation of SPON2 promotes G1 arrest of GC cells. A, B, Flow cytometric analysis of the effects of SPON2 on GC cell cycle progression. Data represent the mean ± SD of three independent experiments, **P < .01. C, Western blot analysis of CCND1, CCNE1 and TP53 expression in recombinant BGC‐823 and SGC‐7901 cell lines and control cells. D, Western blot analysis of CCND1 expression in recombinant BGC‐823 cells (BGC‐823‐shSPON2) transfected with the CCND1‐overexpressing plasmid pcDNA3.1A (−)‐CCND1 and the control plasmid, pcDNA3.1A (−). E, F, Flow cytometric analysis of the cell cycle of BGC‐823‐shSPON2 cells transfected with pcDNA3.1A (−)‐CCND1 or pcDNA3.1A (−), **P < .01. G, *CCND1* mRNA expression in recombinant BGC‐823 and SGC‐7901 cell lines and control cells. #*P* > .05. H, Luciferase reporter‐gene assays were performed to analyse the effects of SPON2 expression on *CCND1* promoter activity in HEK293T and recombinant BGC‐823, and SGC‐7901 cell lines. Relative luciferase activity (mean ± SD) of cells re‐expressing *SPON2* was similar to that of control cells (Student's *t* test, #*P* > .05). This experiment was repeated three times

To identify the mechanism through which *SPON2* influences the proliferation of GC cells, we analysed the cell cycle using flow cytometry. The number of cells in G1 was increased in *SPON2‐*knockouts compared with the control group, while the number of cells in S stage was decreased in *SPON2‐*knockdown cells (Figure 5A,B). CCND1, cyclin E1 (CCNE1) and tumour protein 53 (TP53) are critical in the regulation of G1 arrest. Western blotting data revealed that the levels of CCND1 vs controls in *SPON2*‐knockdown BGC‐823 and SGC‐7901 cells were significantly decreased, while the levels of CCNE1 and TP53 were not significantly different (Figure 5C).

When we used pcDNA3.1A (−) CCND1 to transfect *SPON2*‐knockdown BGC‐823 cells (Figure 5D), we found that CCND1 overexpression reversed the effect of knockdown of *SPON2* expression on the cell cycle (Figure 5E,F). However, qRT‐PCR did not detect a significant difference in CCND1 levels between the *SPON2*‐knockdown and control cells (Figure 5G), and there were no significant effects of *SPON2* expression on the activity of the *CCND1* promoter (Figure 5H).

### 3.6 Down‐regulation of *SPON2* promotes apoptosis

**Figure 6:**
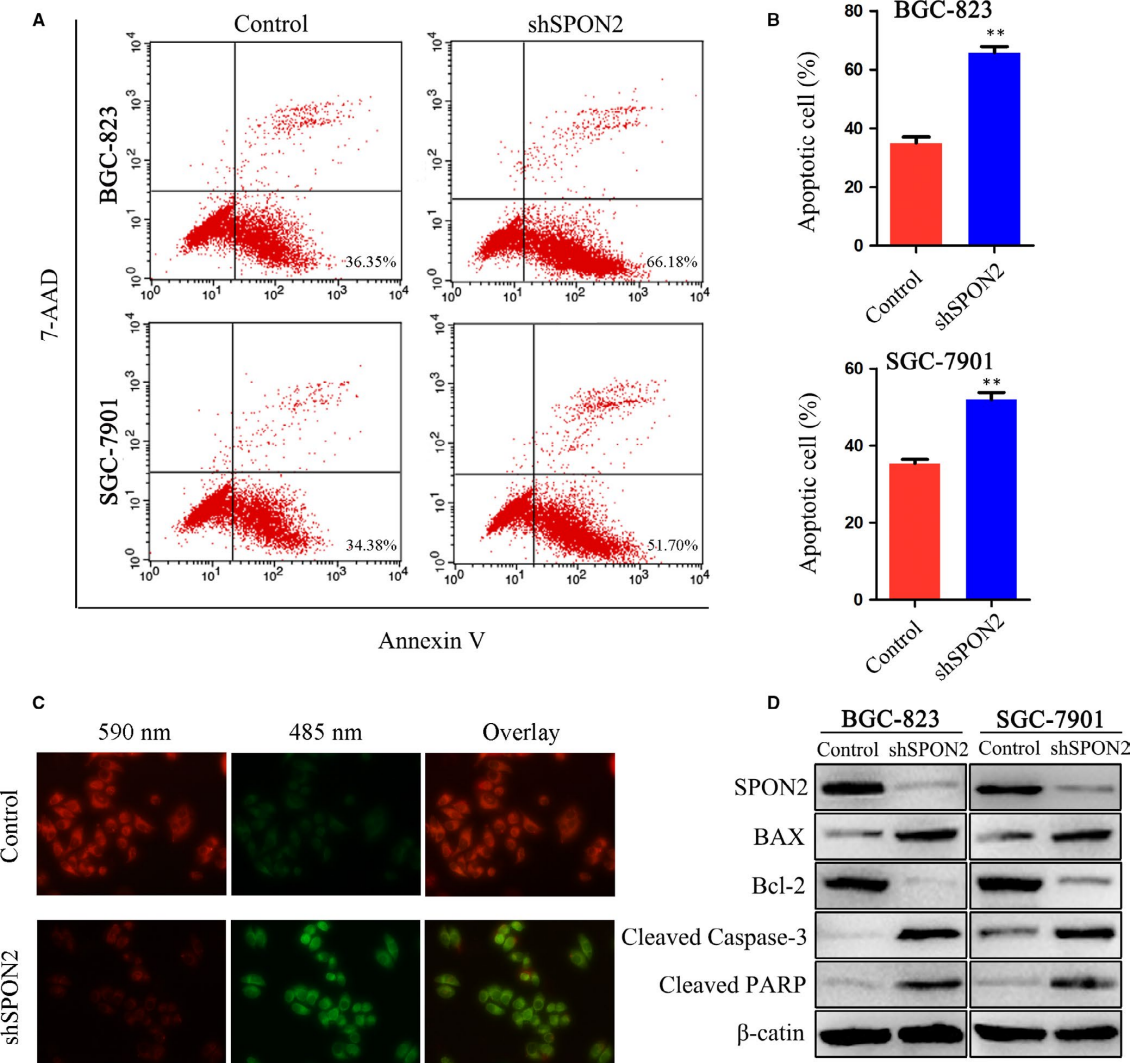
Down‐regulation of *SPON2* promotes apoptosis of GC cells. A, B, Flow cytometric analysis of the effects of SPON2 on apoptosis of GC cells. Data represent as the mean ± SD, **P < .01. C, Representative images of recombinant SGC‐7901 cells and control cells stained with JC‐1. D, Western blot analysis of BAX, BCL2, cleaved CASP3 and cleaved PARP expression in recombinant BGC‐823 and SGC‐7901 cell lines and control cells

Flow cytometry was used to detect apoptosis of GC cells. The rates of apoptosis of *SPON2*‐knockdown BGC‐823 and SGC‐7901 cells were higher compared with those of the controls (Figure 6A,B). We applied JC‐1 fluorescence labelling to detect changes in the mitochondrial membrane potential of GC cells (SGC‐7901). In *SPON2*‐knockdown cells, mitochondrial membrane potential was significantly decreased (Figure 6C). Western blotting revealed that the levels of BCL2 apoptosis regulator (BCL2) in *SPON2*‐knockdown cells were significantly lower compared with those of the controls, while the levels of BCL2‐associated X, apoptosis regulator (BAX), cleaved caspase‐3 (CASP3) and cleaved poly (ADP‐ribose) polymerase 1 (PARP1) were significantly increased (Figure 6D).

### 3.7 *SPON2* promotes the epithelial‐mesenchymal transition (EMT) of GC cells through the ERK1/2 pathway

The EMT mediates tumour metastasis and relapse, and activation of the mitogen‐activated protein kinase (MAPK) pathway contributes to the development and progression of malignant tumours.^26^, ^27^, ^28^, ^29^ To identify the molecular mechanism through which *SPON2* promotes metastasis of GC cells, we performed Western blotting to determine the levels of molecules that act downstream in the MAPK and EMT pathways.

**Figure 7:**
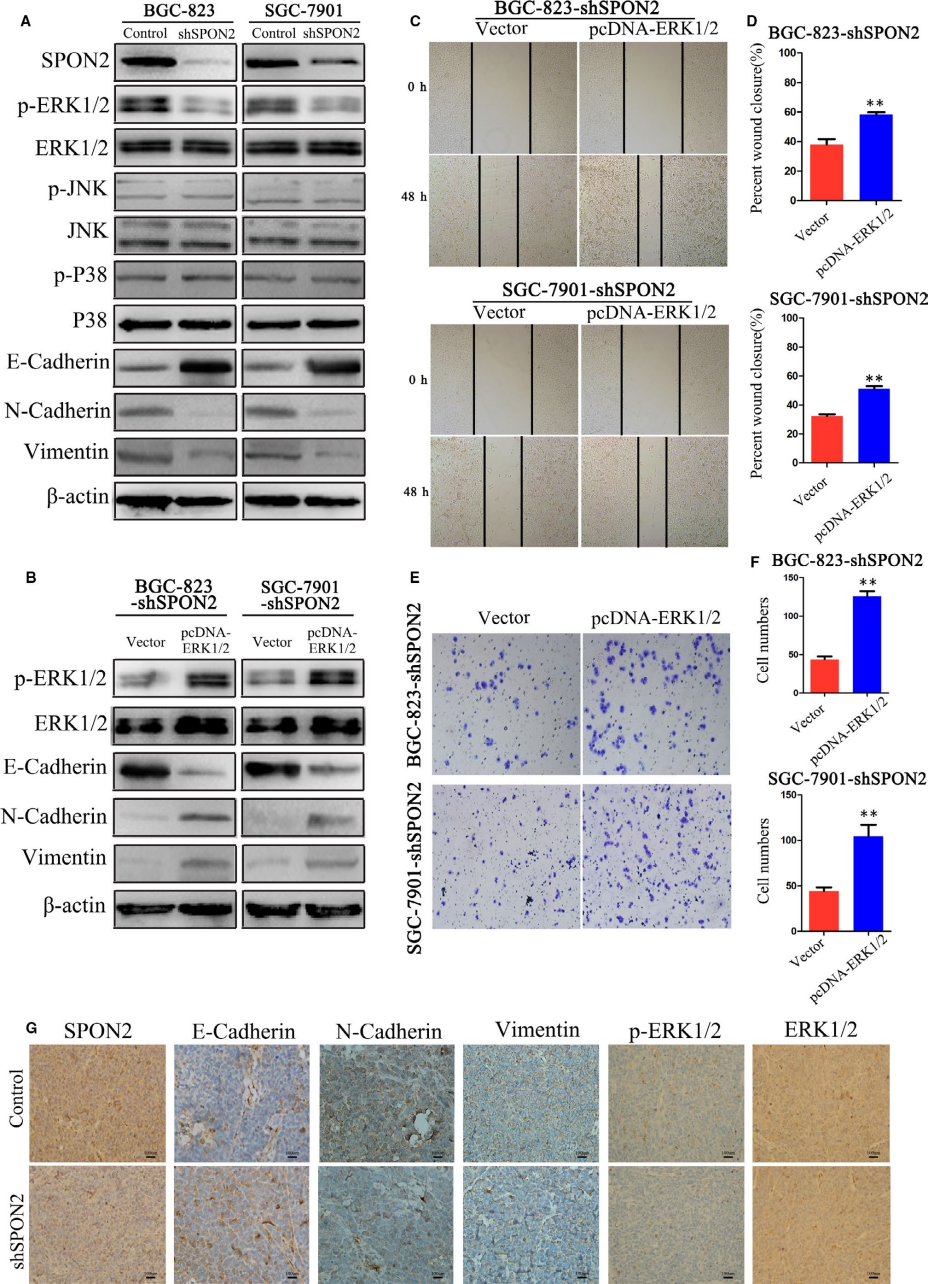
SPON2 promotes the EMT of GC cells through the ERK1/2 signalling pathway. A, Western blot analysis of p‐ERK1/2, ERK1/2, p‐MAPK8, MAPK8, p‐MAPK14, MAPK14, CDH2, VIM and CDH1 expression in recombinant BGC‐823 and SGC‐7901 cell lines and control cells. B, Western blot analysis of p‐ERK1/2, ERK1/2, CDH2, VIM and CDH1 levels in recombinant BGC‐823 (BGC‐823‐shSPON2) and SGC‐7901 (SGC‐7901‐shSPON2) cell lines transfected with an ERK1/2‐overexpressing plasmid (pcDNA‐ERK1/2) or the control vector. C, D, Wound healing and E, F, Transwell invasion assays were performed to detect the migration and invasiveness of BGC‐823‐shSPON2 and SGC‐7901‐shSPON2 cell lines transfected with an ERK1/2‐overexpressing plasmid (pcDNA‐ERK1/2) or a control vector. Data represent the mean ± SD of three independent experiments, **P < .01. G, IHC analysis of SPON2, CDH1, CDH2, VIM, p‐ERK1/2 and ERK1/2 expression in nude mice subcutaneously injection with recombinant SGC‐7901 cells (magnification ×400)

The levels of E‐cadherin (CDH1) in *SPON2‐*knockdown BGC‐823 and SGC‐7901 cells were increased; the levels of phospho(p)‐ERK1/2, cadherin 12 (CDH2, formerly N‐cadherin) and vimentin (VIM) were decreased. Those of ERK1/2, p‐MAPK8 (formerly JNK), MAPK14 (formerly P38), MAPK8 and p‐MAPK14 were unchanged (Figure 7A). When ERK1/2 was overexpressed in *SPON2*‐knockdown cells, the levels of p‐ERK1/2 and ERK increased, those of CDH1 decreased, and those of CDH2 and VIM increased (Figure 7B).

Wound healing and Transwell assays showed that the number of migrating and invading *SPON2‐*knockdown cells that overexpressed ERK was significantly increased compared with those of the controls (Figure 7C‐F). IHC analysis of subcutaneous tumours generated by *SPON2‐*knockout SGC‐7901 cells revealed that compared with the controls, the levels of CDH1 expression increased, those of CDH2, VIM and p‐ERK1/2 decreased, and those of ERK1/2 were unchanged (Figure 7G).

## 4 DISCUSSION

*SPON2* is highly expressed in cancer tissue, although the association with patients' clinicopathological features varies.^13^, ^16^, ^18^ This inconsistency may be explained by tissue‐specific expression, patients' ethnicities, inconsistent assay methods and data analysis methods.^30^, ^31^ Here we used the ONCOMINE cancer microarray database to show that the expression of *SPON2* is significantly higher in GC tissues compared with those in matched para‐tumorous tissues. IHC analysis demonstrated that high expression of *SPON2* was significantly associated with T‐ and N‐staging, OS and DFS, which is consistent with the findings of Jin et al.^32^ Moreover, *SPON2* expression was significantly increased in GC tissues from patients with metastasis and relapse 3 years after surgery, suggesting that *SPON2* overexpression is associated with relapse and metastasis.

The high expression of *SPON2* in tumour tissues is related to the activation of its promoter region. It has been reported that the light‐activated thyroid hormone receptor (TR) directly transactivated *SPON2* transcription via critical TR‐binding sites located within the promoter region of the gene. This is the reason why *SPON2* has increased transcription levels in some types of HCC tissues.^33^ The non‐coding RNAs that are abnormally expressed can also first increase the expression level of its target gene SOX13, and then activating the promoter region of *SPON2* by SOX13 to increase the expression level of *SPON2* in glioma tissues.^34^ There may be similar thyroid hormone and non‐coding RNA expression abnormalities in GC tissues, which requires further study in the future.

Gastric cancer occurs without exhibiting specific clinical manifestations. Therefore, it is important to identify tumour markers with high sensitivity and specificity for clinical screening for early GC. For example, new testing approaches have identified new diagnostic markers for GC, such as circulating tumour cells, cell‐free DNA, non‐coding RNAs and methylated molecules.^35^, ^36^, ^37^

Here we show that the serum levels of *SPON2* were significantly associated with the development and progression of GC. There was no difference in serum SPON2 concentrations between the two groups of patients (CR + PR/PD + SD) before chemotherapy (*P* > .05), but the serum levels of *SPON2* among patients who responded to chemotherapy and underwent radical surgery were significantly decreased, indicating that effective chemotherapy and radical surgery may reduce the secretion of *SPON2* by GC cells. Although the concentration of serum SPON2 cannot predict the sensitivity of GC patients to chemotherapy, a significant reduction in that of SPON2 before and after chemotherapy may indicate the effectiveness of chemotherapy. However, to establish the clinical value of serum levels of *SPON2*, studies of larger numbers of patients are required.

Our research shows that down‐regulation of *SPON2* expression arrested GC cells in G1 and decreased the number of cells in S phase. Further, we discovered that these alterations were mediated by decreased expression of CCND1. CCND1 expression is regulated by mechanisms involving transcriptional activation, mRNA stability, initial translation speed and changes in protein stability.^38^, ^39^ We show here that changes in *SPON2* expression did not affect CCND1 mRNA levels and CCND1 promoter activity, suggesting that down‐regulation of *SPON2* expression may affect CCND1 expression through posttranscriptional control.

The death receptor and mitochondrial pathways represent the major mechanisms controlling apoptosis. We found that down‐regulation of *SPON2* expression did not cause changes in the level of caspase‐8 (CASP8), a component of the death receptor pathway (results not shown). However, down‐regulation of *SPON2* expression reduced the mitochondrial membrane potential of GC cells and significantly increased the levels of cleaved CASP3.

The activation of signalling pathways such as the MAPK cascade contributes to relapse and metastasis of GC. MAPKs are serine/threonine kinases, among which ERK1/2, MAPK8 and MAPK14 are the most widely studied.^40^ In tumour cells, excessive activation of ERK regulates the expression of certain oncogenes, further mediating and amplifying signals during tumour invasion and metastasis.^41^ Our present study shows that high levels of *SPON2* promoted the migration and invasion of GC cells in vivo and in vitro and that p‐ERK1/2 expression in *SPON2*‐knockdown cells, compared with that of the control cells, was significantly decreased, although the levels of ERK1/2, p‐MAPK8, MAPK14, MAPK8 and p‐MAPK14 were not significantly different.

We detected changes in the expression of EMT‐related molecules and found that low levels of *SPON2* inhibited the EMT of GC cells. We presumed therefore that *SPON2* influenced the EMT of GC cells by regulating the activity of the ERK pathway, further affecting migration and invasion. Consistent with this hypothesis, we found that ERK1 overexpression increased the levels of p‐ERK1/2 and activated the ERK pathway, further promoting the EMT. Wound healing and Transwell assays revealed that overexpression of ERK1 reversed the effects of down‐regulating *SPON2* on invasion and migration of GC cells.

In conclusion, high expression of *SPON2* was significantly associated with relapse and metastasis of patients with GC. Serum levels of *SPON2* may be used as a auxiliary diagnostic marker to detect early disease and to evaluate the responses to therapy of patients with GC *SPON2* promoted the EMT of GC cells by activating the ERK pathway. Thus, *SPON2* may serve as a new serological marker for diagnosing GC and as a potential therapeutic target for GC.

## CONFLICT OF INTEREST

The authors declare that there is no conflict of interest.

## AUTHORS' CONTRIBUTIONS

W. Xue and H. Lu conceived the research; W. Xue, H. Lu and Y. Feng designed the experiment; H. Lu and Y. Feng performed the experiments and analysed the data; all authors discussed the manuscript; H. Lu wrote the manuscript; W. Xue, Q. Mao, Y. Feng, Y. Hu, Y. Guo and Y. Liu revised the manuscript; and Q. Mao and W. Xue supervised the project.

## ETHICAL APPROVAL

This study was conducted with the approval of the Institutional Ethical Standards Committee.

## HUMAN RIGHTS STATEMENT AND INFORMED CONSENT

All procedures followed were in accordance with the ethical standards of the responsible committee on human experimentation (institutional and national) and with the Helsinki Declaration of 1964 and later versions. Informed consent or substitute for it was obtained from all patients for being included in the study.

## ANIMAL STUDIES

All institutional and national guidelines for the care and use of laboratory animals were followed.

## Data Availability

The data that support the findings of this study are available on request from the corresponding author. The data are not publicly available due to privacy or ethical restrictions.
