# Supplementary material for: Spondin 2 promotes the proliferation, migration and invasion of gastric cancer cells
Source: J Cell Mol Med. 2019 Nov 5;24(1):98–113. doi: 10.1111/jcmm.14618 (PMC6933360; doi:10.1111/jcmm.14618)
Supplement: Supplementary file 1 [file JCMM-24-98-s001.doc]

**SPON2 promotes the proliferation, migration, and invasion of gastric cancer cells**

Haoming Lu*^1,2^, Ying Feng*^1^, Yilin Hu^1^, Yibing Guo^2^, Yifei Liu^3^, Qinsheng Mao^&1^, and Wanjiang Xue^&1, 2^

**Contents**

Supplementary Figure S1

**Supplementary Figure Legends**


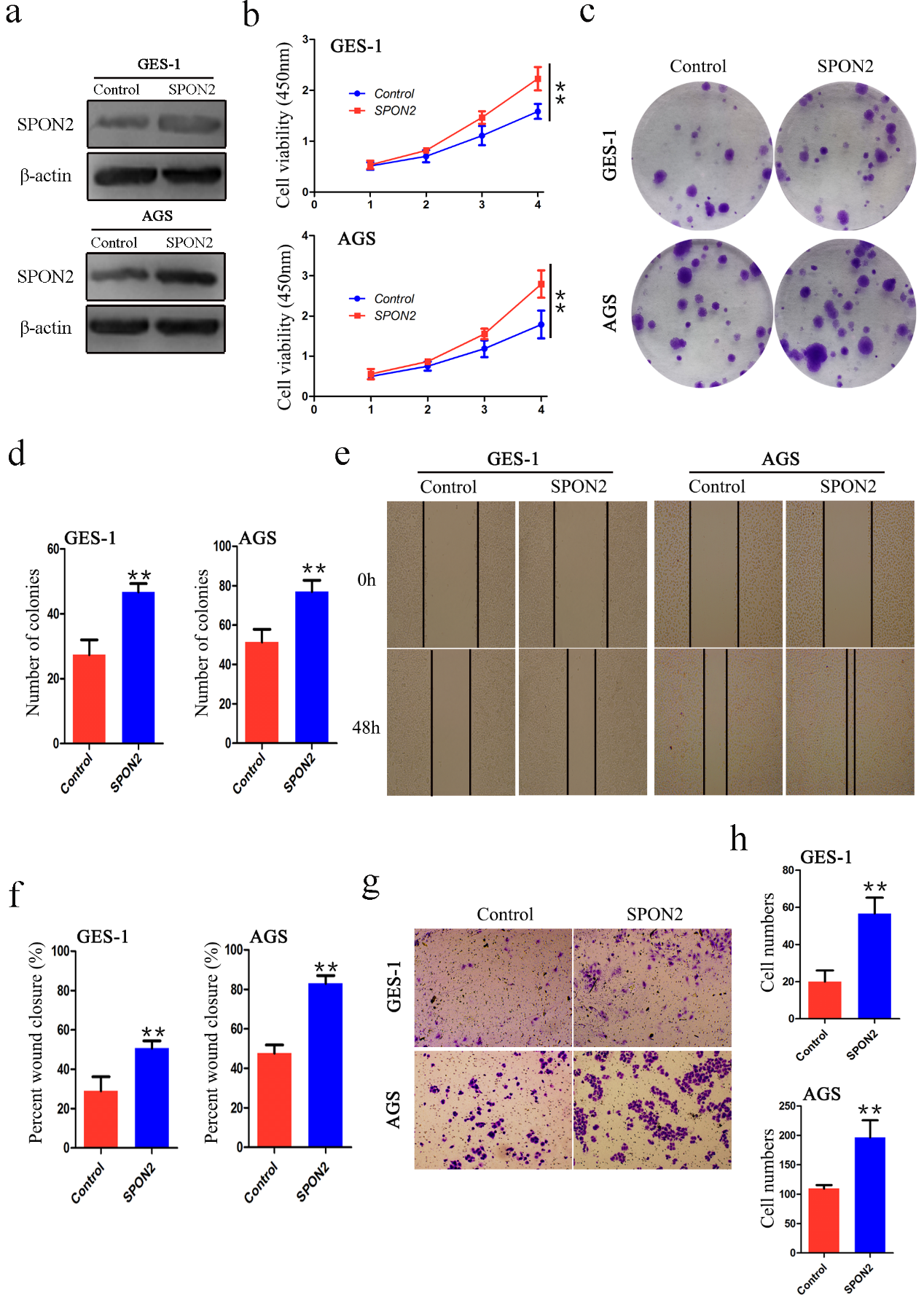


**Supplementary Figure 1. SPON2 promotes GES-1 and AGS proliferation, migration and invasion. a** Western blot assay was used to detect the overexpression of SPON2 in GES-1 and AGS cells. **b** CCK8 assay of the proliferation rates of recombinant GES-1 and AGS cell lines and control cell lines, ***P*<0.01. **c, d** Colony formation assays were performed to detect the effects of SPON2 on the proliferation of recombinant GES-1 and AGS cell lines and control cells. Data represent the mean ± SD of three independent experiments, ***P*<0.01. **e, f** Wound healing and **g, h** Transwell invasion assays were performed to detect the migration and invasiveness of recombinant GES-1 and AGS cell lines and control cells. Data represent the mean ± SD of three independent experiments, ***P*<0.01.
